# SIMVASTATIN as a potential protective strategy against doxorubicin-induced cardiotoxicity

**DOI:** 10.3389/fcvm.2026.1846253

**Published:** 2026-06-09

**Authors:** Barbara Pala, Mariagrazia Piscione, Maria Carmela Di Marcantonio, Francesco Cribari, Roberta Vitale, Thomas Baldi, Ada Popolo, Gabriella Mincione

**Affiliations:** 1 UOC Cardiologia, Ospedale IDI-IRCCS, Roma, Italy; 2 PhD School of Applied Medical-Surgical Sciences, University of Rome Tor Vergata, Roma, Italy; 3 Department of Cardiology, SS. Annunziata Hospital, ASL2 Abruzzo, Chieti, Italy; 4 Department of Cardiology, F. Renzetti Hospital, ASL2 Abruzzo, Lanciano, Italy; 5 Department of Innovative Technologies in Medicine and Dentistry, University “G. d’Annunzio” Chieti-Pescara, Chieti, Italy; 6 Department of Pharmacy, University of Salerno, Fisciano, Italy; 7 Independent Researcher, Roma, Italy

**Keywords:** cardioprotection, cholesterol, doxorubicin-induced cardiotoxicity, electrocardiographic abnormalities, simvastatin

## Abstract

**Background:**

Doxorubicin-induced cardiotoxicity (DIC) represents a major limitation in oncology, leading to ventricular dysfunction and long-term morbidity. Lipophilic statins, such as simvastatin, exert pleiotropic effects beyond cholesterol lowering, including antioxidant and anti-inflammatory actions, which may confer cardioprotection.

**Methods:**

We retrospectively analyzed 80 oncology patients treated with anthracycline-based chemotherapy. Clinical, biochemical, and electrocardiographic (ECG) data were collected at baseline and after completion of chemotherapy or during follow-up. Early chemotherapy-related cardiac dysfunction was assessed using ECG markers, including QTa/QTc prolongation and T-wave flattening. Reduced ejection fraction (HFrEF) was defined as left ventricular ejection fraction (LVEF) < 50%. Patients were stratified according to exposure to simvastatin therapy versus no statin treatment. Associations between statin use and cardiac outcomes were evaluated using adjusted regression models; additional propensity score–based weighting analyses were performed to account for potential baseline differences between groups.

**Results:**

Seven patients developed HFrEF. Among patients with preserved LVEF (>60%), 25 developed new ECG abnormalities, whereas 39 maintained normal ECG findings. Statin therapy was strongly associated with protection against ECG alterations: 23 of 25 patients with ECG changes were not receiving statins, while 33 of 39 patients without abnormalities were statin users. Statin-treated patients showed significantly smaller declines in LVEF (ΔLVEF −1.7% vs. −8.0%, *p* = 0.0017) and reduced prolongation of ventricular repolarization intervals (ΔQT and ΔQTc) compared with non-users. In adjusted analyses, simvastatin exposure remained independently associated with preservation of systolic function and attenuation of QT/QTc prolongation. Statin-treated patients also exhibited lower total and low-density lipoprotein (LDL) cholesterol levels, consistent with expected pharmacologic effects. No clinically relevant differences were observed in atrioventricular or intraventricular conduction parameters. Propensity score–weighted analyses confirmed the robustness of the association between statin therapy and reduced risk of electrocardiographic abnormalities.

**Conclusion:**

Statin therapy was associated with a lower incidence of early electrocardiographic abnormalities and attenuation of subclinical cardiac dysfunction in patients treated with doxorubicin. These findings suggest that lipophilic statins may mitigate early electrophysiological remodeling and preserve ventricular function during anthracycline therapy, supporting a potential cardioprotective role beyond lipid lowering.

## Introduction

Advances in cancer therapy have markedly improved patient survival over recent decades (1). However, many effective chemotherapeutic agents remain limited by significant cardiovascular toxicity. Among these, anthracyclines, particularly doxorubicin (Doxo), are widely used in the treatment of lymphomas, sarcomas, and breast cancer, but are associated with a substantial risk of cardiotoxicity. Doxo exerts its antineoplastic effects primarily through inhibition of deoxyribonucleic acid (DNA) topoisomerase II, leading to DNA damage in rapidly proliferating tumor cells (2). Despite its efficacy, up to 20%–25% of exposed patients may develop cardiac complications, reducing its long-term clinical use (3).

Clinically, doxorubicin-induced cardiotoxicity (DIC) encompasses a broad spectrum of manifestations, including reductions in left ventricular ejection fraction (LVEF), ventricular remodeling, arrhythmias, and progression to heart failure (HF), which may be irreversible and life-threatening (4). The risk of cardiotoxicity is dose-dependent, increasing substantially with cumulative doses exceeding 400–700 mg/m^2^ (5, 6). At the molecular level, DIC is driven by excessive production of reactive oxygen species (ROS), mitochondrial dysfunction, altered calcium homeostasis, and activation of apoptotic pathways (7). Cardiomyocytes are particularly susceptible due to high oxidative metabolism and limited antioxidant capacity (8).

Statins, primarily prescribed as inhibitors of *β*-Hydroxy *β*-methylglutaryl-CoA (HMG-CoA) reductase for lipid lowering, exert multiple pleiotropic effects that may be beneficial in the setting of chemotherapy-induced cardiotoxicity. Lipophilic statins such as simvastatin and atorvastatin demonstrate antioxidant, anti-inflammatory, and endothelial-protective properties (9, 10). These agents readily penetrate cell membranes, allowing them to exert cholesterol-independent effects in vascular and myocardial tissue, including attenuation of ROS generation, inhibition of Nuclear Factor Kappa-light-chain-enhancer of activated B cells (NF-*κ*B) signaling, and prevention of maladaptive hypertrophy (11, 12). Abd et al, have demonstrated that simvastatin mitigates doxorubicin-induced cardiotoxicity in rats by reducing oxidative stress, lipid peroxidation, and mortality, while preserving myocardial antioxidant defenses (13). Randomized recent clinical trial (as an example STOP-CA trial—Atorvastatin for the Prevention of Anthracycline-Related Cardiac Dysfunction) (14–16) and observational studies have suggested that statin therapy may mitigate anthracycline-associated declines in LVEF and reduce the incidence of HF (17–20). In addition, statins have been reported to exert anti-neoplastic effects, potentially influencing tumor proliferation and metastasis (21, 22). Specifically, simvastatin has been shown to suppress epithelial–mesenchymal transition and extracellular matrix degradation, thereby reducing cancer cell migration, reported in recent manuscript (23).

Lipophilic statins, such as simvastatin, were specifically selected because of their well-established cholesterol-independent effects on myocardial and vascular tissues (24). In addition, simvastatin is generally considered a manageable, moderate- to low-intensity statin, which enhances its clinical applicability. Should its cardioprotective and pleiotropic effects be confirmed, this therapeutic approach may be particularly suitable for more vulnerable and elderly populations, often characterized by multiple comorbidities and polypharmacy (25). This consideration is especially relevant in the contemporary oncological setting, where cancer is increasingly managed as a chronic condition and the number of long-term cancer survivors continues to rise.

Moreover, higher expression levels of HMG-CoA reductase (HMGCR) were associated with significantly shorter median overall survival, in lymphoma. These findings support a biologically relevant role of cholesterol biosynthesis and its downstream signaling pathways in tumor progression and provide a mechanistic rationale for exploring statin-based therapeutic strategies in this setting (26, 27).

Despite these promising observations, the role of statins as cardioprotective agents in patients exposed to anthracyclines remains insufficiently explored and debated (28, 29). Recent comprehensive reviews have further emphasized the emerging role of lipid-lowering therapies, particularly statins, as potential cardioprotective agents in anthracycline-treated patients, highlighting their antioxidant, anti-inflammatory, and mitochondrial-stabilizing properties, as well as the need for clinically oriented validation studies in real-world cardio-oncology settings (30). Recent evidence has highlighted the potential value of ECG markers as early indicators of DIC. In particular, Kinoshita et al. (31) demonstrated that QTa prolongation and T-wave flattening may precede overt systolic dysfunction and serve as early markers of chronic anthracycline cardiotoxicity. Mechanistic analyses further suggest that a QTa increase greater than 10% from baseline or an absolute prolongation of ≥ 30–40 ms should be considered clinically relevant warning signs (27, 31). These findings underscore the importance of integrating ECG parameters with biochemical and echocardiographic data when evaluating cardioprotective strategies.

The present study aims to investigate whether statin therapy, specifically simvastatin, is associated with cardioprotective effects in oncology patients treated with doxorubicin, with particular focus on lipid profile, left ventricular systolic function, and early ECG alterations including *P*-wave duration, QTa, QTc, and T-wave morphology.

## Material and methods

### Study population

This retrospective observational study was conducted at the Clinical Oncology Unit of the “Santissima Annunziata” Hospital in Chieti (Italy) and included patient treated with anthracycline-based chemotherapy regimens containing doxorubicin. A total of 80 consecutive oncology patients were initially screened. This study was approved by the local ethics committee on October 2, 2025.

The study population was predominantly composed of patients with breast cancer (approximately 72%), while the remaining cases included lymphomas (18%) and soft tissue sarcomas (10%), reflecting the routine clinical use of anthracycline-based regimens in these malignancies.

All patients received a total of six chemotherapy cycles consisting of doxorubicin at a dose of 60 mg/m^2^ and cyclophosphamide at a dose of 600 mg/m^2^, administered every three weeks, in accordance with current clinical practice and available literature (32).

Concomitantly, a subset of patients received taxane-based chemotherapy and trastuzumab as part of adjuvant treatment.

Given the established cardiotoxic effects of trastuzumab, its use was considered a potential confounder. However, in this cohort, trastuzumab was administered only after completion of anthracycline therapy and after the ECG follow-up time point (T1), thereby minimizing its influence on the electrocardiographic and echocardiographic outcomes analyzed. Radiotherapy was administered after completion of the study period. Eligible patients who had not previously received trastuzumab were treated with this agent following the end of chemotherapy.

Patients were eligible for inclusion if they had received 6 cycles of doxorubicin-containing chemotherapy within the previous two years and if complete clinical data were available, including laboratory tests and standard 12-lead electrocardiograms performed both before the initiation of chemotherapy (T0) and after 6 cycles of therapy (T1).

Patients were excluded if they had a prior history of acute myocardial infarction, the presence of a permanent pacemaker or other implantable cardiac devices, acute cardiovascular events or hemodynamic instability at the time of data collection, or if they were receiving statins other than simvastatin These criteria were applied to minimize confounding factors related to pre-existing cardiac conduction abnormalities or heterogeneous lipid-lowering therapies.

Patients were stratified according to statin exposure: those receiving simvastatin at the 20 mg daily were included in the statin group, while patients not receiving any statin therapy served as the control group.

Demographic variables included age and sex. Clinical, biochemical, and electrocardiographic data were collected retrospectively from medical records at both time points.

### Electrocardiographic assessment

Standard resting 12-lead surface electrocardiograms were obtained for all patients using a conventional ECG recording system, with a paper speed of 25 mm/s and a standard calibration of 10 mm/mV. ECGs were recorded at two predefined time points: before initiation of Doxo-based chemotherapy (T0) and after 6 cycles of Doxo- treatment (T1).

Electrocardiographic analysis focused on parameters reflecting atrio-ventricular conductiona and ventricular repolarization. The following variables were measured: PR interval, QT interval, QTa interval, corrected QT interval (QTc), and T-wave morphology. QT and QTa intervals were measured using the tangent method by drawing a tangential line at the maximal downslope of the terminal portion of the T wave, and measurements were averaged over three consecutive cardiac cycles with minimal RR variability (33). QT measurements were preferentially obtained in leads II and V5, as recommended for assessment of ventricular repolarization. QTc was calculated using the Freidericia's correction formula (34). All ECG measurements were performed manually by an experienced cardiologist blinded to clinical data, statin exposure, and patient outcomes.

T-wave morphology was assessed qualitatively and quantitatively. T-wave height was quantified as an index of ventricular repolarization. Following identification of the T-wave termination by the tangent method, the vertical distance from the baseline to the maximal T-wave peak was measured (35). T-wave flattening was defined by a reduction in T-wave amplitude, measured as the vertical distance from the isoelectric baseline to the maximal T-wave peak after identification of the T-wave termination by the tangent method.

ECG abnormalities were defined as the development of QTa or QTc prolongation and/or T-wave flattening at follow-up. These changes were analyzed in relation to statin exposure and the occurrence of Doxo-induced cardiac dysfunction.

### Symptoms, echocardiography and blood examination

Laboratory assessments comprised hemoglobin, hematocrit, fasting plasma glucose, total cholesterol, high-density lipoprotein (HDL) cholesterol, low-density lipoprotein (LDL) cholesterol, and triglycerides.

HF status was estimated using both clinical and echocardiographic information. HF status was inferred from the institutional database, which classified patients as having developed or not sign and symptoms of HF during follow-up. In addition, LVEF was evaluated echocardiographically and categorized as normal (LVEF > 60%), mildly reduced (LVEF 50%–60%), or reduced (LVEF < 50%). This dual approach allowed integration of clinical diagnosis with objective measures of LVEF (36).

Left ventricular ejection fraction (LVEF) was assessed using two-dimensional echocardiography based on the Simpson's biplane method, with additional three-dimensional measurements when available (35).

Additional echocardiographic parameters, including left ventricular end-diastolic volume (LVEDV), left ventricular end-systolic volume (LVESV), stroke volume, and myocardial strain analysis, were not systematically available because of the retrospective design and the heterogeneity of echocardiographic examinations performed during routine clinical practice.

### Statistical analysis

Statistical analyses were performed using Python (version 3.11) with the pandas, numpy, statsmodels, scipy, and scikit-learn libraries. Analyses were conducted on patients with complete paired measurements at baseline (T0) and follow-up (T1).

Continuous variables were summarized as mean ± standard deviation or median with interquartile range, as appropriate. Categorical variables were reported as counts and percentages. For all continuous parameters measured at both time points, absolute changes were calculated as Δ = T1 − T0.

Baseline characteristics were compared between the statin and no statin groups using the Wilcoxon rank-sum test for continuous variables and the Pearson χ^2^ test or Fisher's exact test for categorical variables, according to expected cell count.

The association between statin therapy and the occurrence of ECG abnormalities, defined as QTa/QTc prolongation and/or T-wave flattening, was first assessed by univariate analysis using χ^2^ or Fisher's exact test. Subsequently, a logistic regression model was applied to estimate odds ratios (ORs) with 95% confidence intervals (95% CI).

Longitudinal changes in lipid parameters and PR interval between baseline (T0) and follow-up (T1) in statin-treated patients were analyzed using the Welch two-sample t-test or equivalent non-parametric tests when appropriate.

Given the limited sample size and partial imbalance in baseline covariates, an exploratory multi-model analytical strategy was adopted.

The dataset underwent systematic quality control and harmonization prior to analysis. Column names were standardized, and all numerical variables were converted to floating-point format. Missing values were handled by coercing non-numeric entries to NaN using “pandas.to_numeric” with the argument “errors='coerce'”. For variables measured at baseline (T0) and follow-up (T1), Δ variables were automatically generated to represent within-subject changes. Internal consistency between related measurements was verified (e.g., concordance between the sign of ΔQTc and the observed T0–T1 difference). Categorical variables were recoded as binary (0/1) for statistical analyses.

For continuous outcomes (e.g., ΔLVEF%, ΔQT, ΔQTc), ordinary least squares (OLS) regression with heteroscedasticity-consistent (HC3) robust standard errors was applied. Robust linear models with Huber loss function were additionally estimated to assess sensitivity to outliers.

Binary endpoints, including the occurrence of new ECG abnormalities and composite cardiac outcomes, were analyzed using logistic regression. Penalized logistic models were also considered to reduce small-sample bias and quasi-separation.

To assess the stability of effect estimates, non-parametric bootstrap resampling (1,000 iterations) was performed for key outcomes, generating empirical 95% confidence intervals for regression coefficients and odds ratios.

To further address confounding due to non-random statin exposure, a propensity score for simvastatin treatment was estimated using baseline demographic, echocardiographic, ECG, and lipid variables. Inverse probability of treatment weights (IPTW) was derived and stabilized, with trimming at the 1st and 99th percentiles. Covariate balance before and after weighting was assessed using standardized mean differences.

Weighted least squares (WLS) and weighted generalized linear models (GLS) were then fitted to estimate marginal treatment effects under the IPTW framework. Multicollinearity was evaluated using variance inflation factors (VIF < 3 for all models). All statistical tests were two-sided, and *p*-values < 0.05 were considered statistically significant.

## Results

Of the 80 patients initially included, 71 patients were eligible for analysis, with paired ECG recordings at baseline (T0) and follow-up (T1). Nine patients were excluded due to atrial fibrillation or major conduction disturbances. Baseline demographic, clinical, biochemical, and electrocardiographic characteristics are summarized in Table 1.

**Table 1:** Baseline demographic, clinical, biochemical, and electrocardiographic characteristics according to statin use.

Characteristic	Statin user *N* = 39	Non-Statin user *N* = 32	*p*-value
Age, years (mean ± SD)	56.95 ± 11.52	60.03 ± 10.38	0.21
Sex			0.16
Female sex, *n* (%)	26 (66.67)	26 (66.67)	
Male sex, *n* (%)	13 (33.33)	6 (18.75)	
BMI, kg/m^2 (mean ± SD)	24.00 ± 5.00	23.00 ± 4.50	0.64
Comorbidities			
Type 2 diabetes mellitus, *n* (%)	8 (20.51)	6 (18.75)	0.83
Hypertension, *n* (%)	2 (5.13)	2 (6.25)	0.82
Thyroid dysfunction, *n* (%)	15 (38.46)	12 (37.50)	0.94
Autoimmune disease, *n* (%)	12 (30.77)	10 (31.25)	0.96
Chronic Kidney disease, *n* (%)	1 (2.56)	1 (3.13)	0.90
Malignancy Characteristics			
Breast cancer *n* (%)	28 (71.79)	23 (71.88)	0.99
Hematologic malignancies, *n* (%)	6 (15.38)	6 (18.75)	0.69
Non-Hematologic malignancies, other solid tumors, *n* (%)	5 (12.82)	3 (9.38)	0.61
Concomitant oncologic treatments			
Trastuzumab, *n* (%)	20 (51.30)	14 (43.80)	0.48
Other targeted therapiesa, *n* (%)	7 (17.90)	5 (15.60)	0.78
Lab text			
HGB (g/dL)	13.17 ± 1.73	13.72 ± 1.48	0.09
HCT (%)	36.45 ± 1.67	37.25 ± 1.95	0.05
Glycaemia (mg/dL)	86.69 ± 10.06	87.16 ± 11.09	0.83
Total cholesterol (mg/dL)	207.36 ± 32.93	203.68 ± 34.66	0.70
HDL cholesterol (mg/dL)	50.27 ± 7.52	51.49 ± 9.32	0.55
LDL cholesterol (mg/dL)	130.82 ± 21.87	129.97 ± 23.82	0.79
Triglycerides (mg/dL)	150.68 ± 54.87	151.62 ± 53.56	0.84
Electrocardiographic parameters			
QT (ms)	392.62 ± 15.20	393.34 ± 17.57	0.79
QTC (ms)	422.56 ± 22.59	426.47 ± 21.60	0.71
QTA (ms)	357.26 ± 16.46	358.31 ± 18.96	0.71
PR (ms)	161.50 ± 5.33	159.90 ± 6.46	0.68

Continuous variables are reported as mean ± standard deviation (SD), and categorical variables as number (percentage). Comparisons between groups were performed using the Wilcoxon rank-sum test for continuous variables and Pearson's chi-squared test for categorical variables. BMI, body mass index; HGB, Hemoglobin; HCT, hematocrit; HDL, high-density lipoprotein; LDL, low-density lipoprotein.

Mean ± SD; *n* (%).

Wilcoxon rank sum test; Pearson's Chi-squared test.

a Includes agents such as methotrexate or monoclonal antibodies other than trastuzumab.

During follow-up, seven patients developed overt heart failure, defined as a left ventricular ejection fraction (LVEF) < 50% at T1. Although representing a minority of the cohort, these cases highlight the potential severity of Doxo -induced cardiotoxicity.

Among the remaining patients with preserved systolic function (LVEF > 60%), ECG findings were further analyzed to identify early markers of cardiac involvement.

### Electrocardiographic abnormalities

Among patients with preserved LVEF, 25 individuals developed new ECG abnormalities between T0 and T1, whereas 39 patients maintained stable ECG patterns throughout follow-up.

The observed ECG abnormalities were mainly characterized by QTa and QTc prolongation and an increased prevalence of T-wave flattening, consistent with previously described early markers of chemotherapy-related cardiac dysfunction (31).

The distribution of ECG abnormalities was strongly associated with statin therapy. Specifically, 23 of the 25 patients developing ECG abnormalities were not receiving statins, whereas 33 of the 39 patients without ECG abnormalities were statin users.

**Figure 1:**
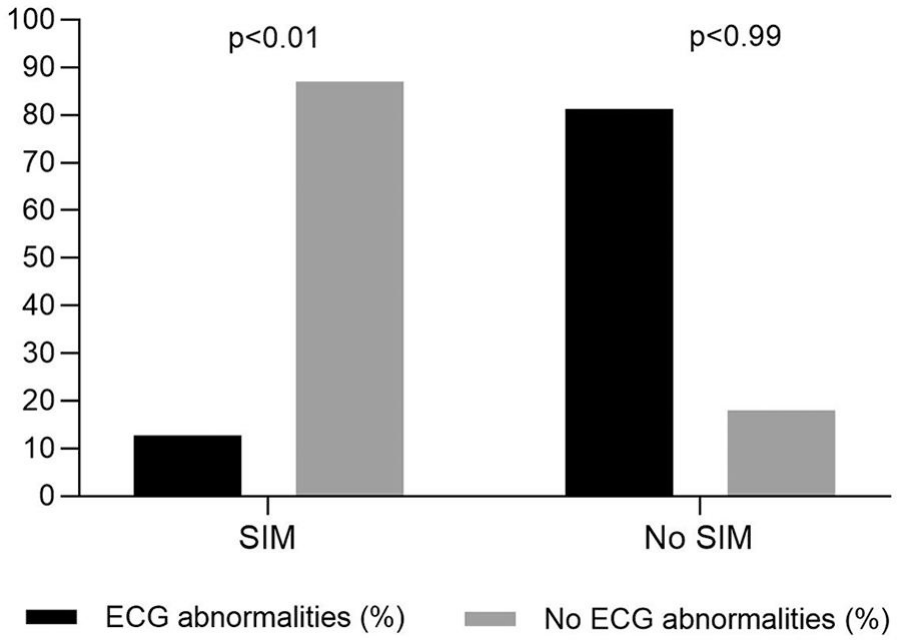
Statin therapy, ECG abnormalities and left ventricular function. Statin-treated patients showed a markedly lower incidence of ECG abnormalities compared with those not receiving statins [12.8% [5/39] vs. 81.3% [26/32], *p* < 0.001]. Most statin users maintained normal ECG patterns, whereas repolarization abnormalities were highly prevalent in the no-statin group.

Statin therapy was associated with a significantly lower incidence of ECG abnormalities compared with no statin treatment [12.8% [5/39] vs. 81.3% [26/32], *p* < 0.001] (Figure 1). In contrast, the occurrence of left ventricular systolic dysfunction (LVEF < 50%) did not differ significantly between groups (10.3% vs. 9.4%, *p* = 0.999). The absence of statistical significance for LVEF outcomes likely reflects the limited number of events, supporting the concept that ECG alterations may precede measurable systolic dysfunction.

Patients treated with statins exhibited lower mean total cholesterol and LDL cholesterol levels compared with non-users, both at baseline and at follow-up (*p* < 0.001). Although the primary focus of this analysis was not longitudinal lipid variation, statin-treated patients showed a significant reduction in total cholesterol and LDL levels over time (the median change was −8.9 mg/dL in the no-SIM group and −19.2 mg/dL in the SIM group (*β*_SIM = −8.1, 95% CI −15.4 to −0.7; *p* = 0.0311), consistent with the expected pharmacological effects of statin therapy. HDL cholesterol and triglyceride levels did not differ significantly**.**

The PR interval was specifically evaluated to assess potential effects of chemotherapy and statin therapy on atrioventricular conduction. In statin-treated patients, the PR interval remained stable between T0 and T1 (162.7 ± 15.1 ms vs. 162.5 ± 15.3 ms; *p* = 0.964). Similarly, when considering the entire cohort, no significant change in PR interval was observed over time (161.3 ± 15.4 ms at T0 vs. 162.1 ± 15.4 ms at T1; *p* = 0.765).

### Effect of simvastatin on left ventricular function

Beyond descriptive comparisons, advanced inferential and causal-inference analyses were performed to further evaluate the association between simvastatin therapy and doxorubicin-induced cardiac alterations.

The change in left ventricular ejection fraction (ΔLVEF%, T1-T0) was significantly attenuated in patients receiving simvastatin. Mean ΔLVEF% was −1.7% in the statin group compared with −8.0% in non-statin patients (*p* = 0.0017).

**Figure 2:**
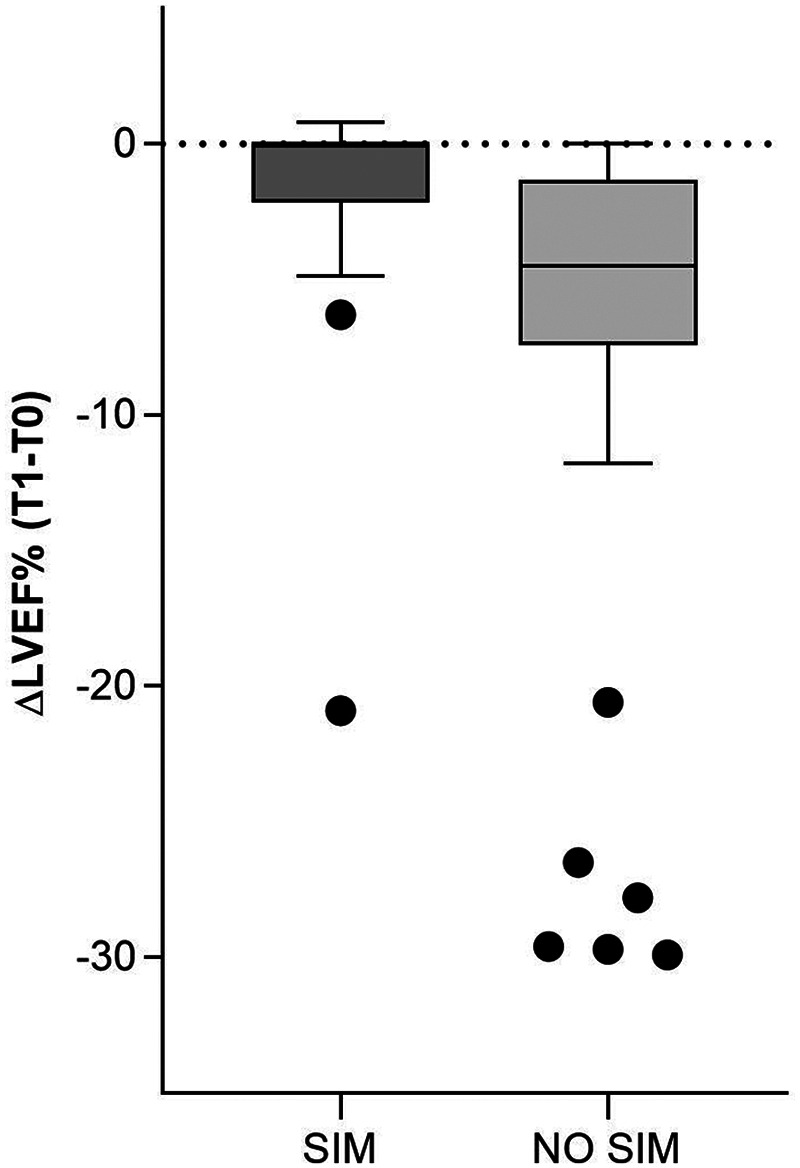
Change in left ventricular ejection fraction (ΔLVEF%, T1–T0) according to simvastatin use. Box plots compare patients treated with simvastatin (SIM) and those not receiving simvastatin (no-SIM). The median ΔLVEF was −0.1% in the SIM group and −4.5% in the no-SIM group. Linear regression analysis showed a significant association between simvastatin use and a more favorable change in LVEF (*β*_SIM = + 6.19, 95% CI 2.59 to 9.79; *p* = 0.000762). Boxes represent the interquartile range, the horizontal line indicates the median, whiskers denote the range excluding outliers, and circles indicate outliers.

In adjusted ordinary least squares (OLS) models with robust variance estimation, simvastatin exposure remained independently associated with preservation of systolic function (*β*_SIM = + 6.19, *p* < 0.001). These findings were confirmed using robust linear models and bootstrap resampling, with consistent effect size and direction (Figure 2).

### Electrocardiographic interval dynamics

**Figure 3:**
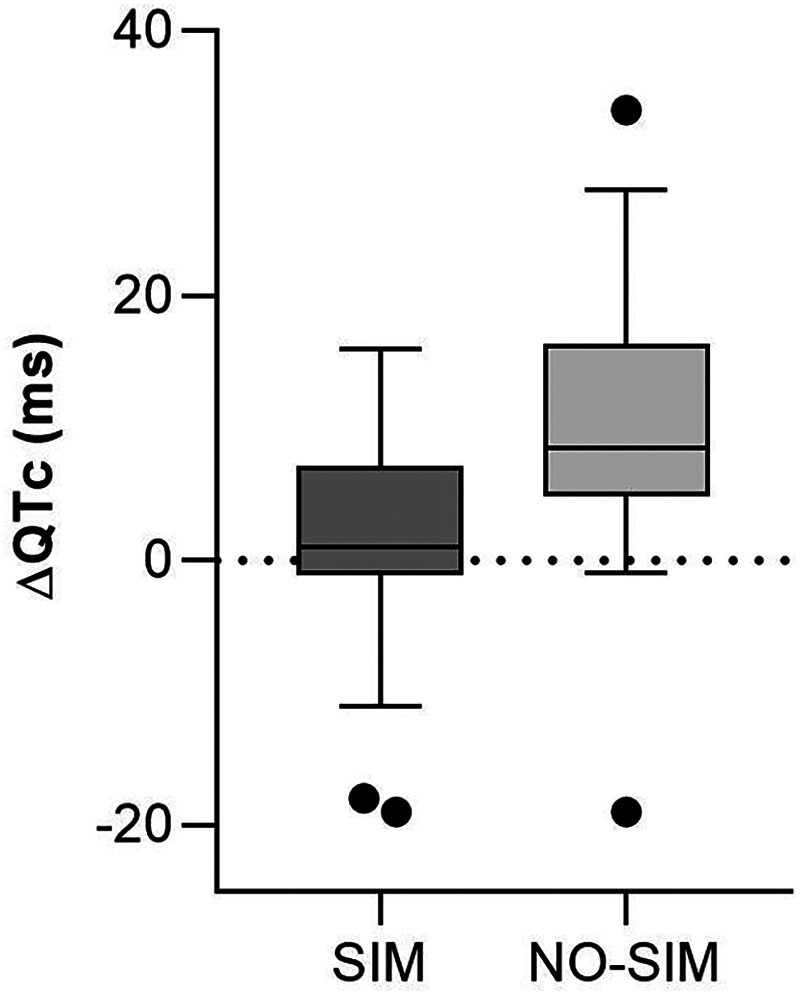
Changes in ventricular repolarization parameters during doxorubicin-based chemotherapy according to statin exposure. Simvastatin-treated patients exhibited significantly smaller increases in QT and corrected QT (QTc) intervals compared with non-statin patients. In adjusted models, simvastatin use was associated with reduced QTc prolongation (*β*_SIM = −8.8 ms, *p* = 2.2 × 10⁻⁵) and QT prolongation (*β*_SIM = −9.7 ms, *p* = 3.8 × 10⁻⁹). No significant differences were observed for PR or QRS interval changes, indicating a selective effect on ventricular repolarization.

Simvastatin therapy was associated with significantly smaller increases in ventricular repolarization intervals. In adjusted models, ΔQTc and ΔQT were markedly reduced in statin-treated patients (*β*_SIM = −8.8 ms for QTc, *p* = 2.2 × 10⁻⁵; *β*_SIM = −9.7 ms for QT, *p* = 3.8 × 10⁻⁹) (Figure 3).

No significant associations were observed for changes in PR or QRS intervals, indicating that the protective effect was specific to ventricular repolarization rather than atrioventricular or intraventricular conduction as summarized in Table 2.

**Table 2:** Mean changes in electrocardiographic parameters (Δ, T1–T0) according to simvastatin use.

Parameter	Δ Mean SIM	Δ Mean no-SIM	β_SIM (adjusted)	*p*-value
ΔQTc (ms)	+1.5	+9.6	−8.8	2.2 e^−05^
ΔQT (ms)	+5.2	+14.5	−9.7	3.8 e^−09^
ΔQR (ms)	+2.1	+3.7	−1.6	n.s.
ΔQRS (Ms)	+1.4	+1.8	−0.4	N.S.

The table reports the mean variation in QTc, QT, PR, and QRS intervals in patients treated with simvastatin (SIM) and in those not receiving simvastatin (no-SIM). The adjusted regression coefficient for simvastatin use (β_SIM) and corresponding *p*-values are shown. Statistically significant associations were observed for ΔQTc and ΔQT, whereas no significant differences were found for ΔPR and ΔQRS (n.s., not significant).

### Composite ECG endpoints

When ECG alterations were analyzed as binary outcomes, simvastatin therapy was strongly associated with a lower probability of developing new ECG abnormalities. The odds ratio for the occurrence of any new ST–T alteration (“any_st_change”) was 0.09 (95% CI 0.02–0.36; *p* = 0.0006).

**Figure 4:**
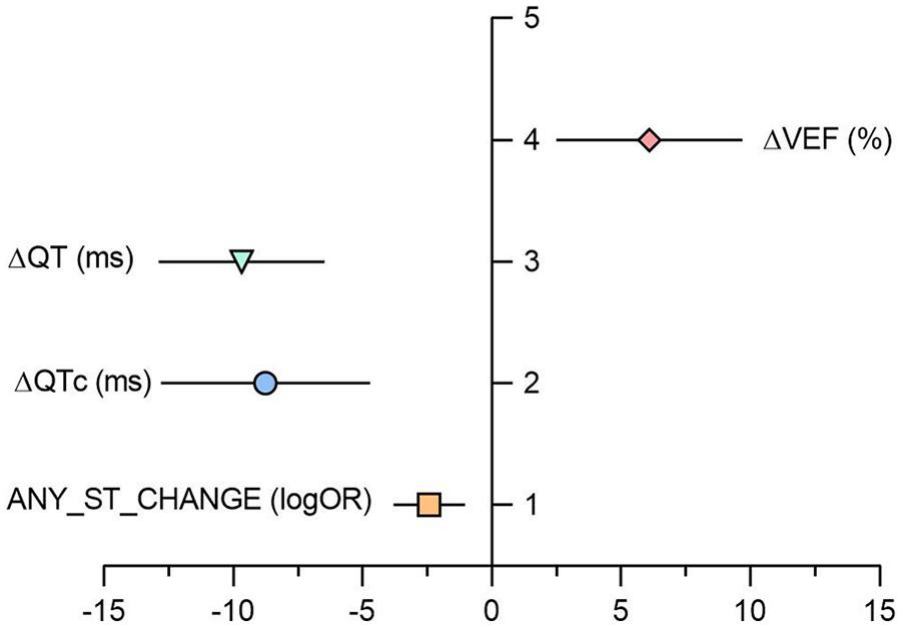
Forest plot summarizing the estimated effects of simvastatin therapy on functional, electrocardiographic, and composite cardiac outcomes. Regression coefficients (*β*) are shown for continuous outcomes and odds ratios (OR) for binary endpoints, with 95% confidence intervals. Simvastatin therapy was associated with preservation of LVEF and attenuation of QT and QTc prolongation, along with a reduced likelihood of ECG abnormalities. Points represent effect estimates with 95% confidence intervals.

Similarly, simvastatin was associated with a significant reduction in the composite endpoint combining ECG abnormalities and relevant LVEF decline (OR = 0.18, 95% CI 0.05–0.62; *p* = 0.006) (Figure 4).

## Discussion

This retrospective cohort analysis provides further evidence that statin therapy may exert a protective role against anthracycline-induced cardiotoxicity at both functional and electrophysiological levels.

Moreover, while previous relevant reviews have synthesized mechanistic and experimental data supporting statin cardioprotection in anthracycline therapy (30).

Baseline demographic, clinical, and electrocardiographic characteristics were overall balanced between groups.

A formal risk stratification according to HFA-ICOS criteria could not be performed due to the lack of serial biomarker data (e.g., troponins and natriuretic peptides) (27). As a result, the study population should be considered heterogeneous in terms of baseline risk for anthracycline-induced cardiotoxicity.

In our population, the incidence of overt heart failure was in line with previous reports describing doxorubicin-related left ventricular dysfunction in approximately 20%–30% of exposed patients (5). Although heart failure occurred in a limited number of cases, nearly half of these patients were not receiving statin therapy, suggesting that the absence of cardioprotective treatment may contribute to increased vulnerability to chemotherapy-related myocardial injury.

Importantly, the preservation of LVEF observed in statin-treated patients remained consistent after adjustment for baseline cardiovascular risk factors and after causal weighting. The magnitude of the effect—approximately a 6% relative difference in LVEF decline—appears biologically meaningful and aligns with experimental evidence linking statins to mitigation of oxidative stress, mitochondrial dysfunction, and cardiomyocyte apoptosis.

These findings are consistent with previous clinical observations showing that chronic statin therapy may attenuate early declines in left ventricular ejection fraction in patients receiving anthracycline treatment (37).

Although the number of patients developing overt systolic dysfunction was limited, 2 out of 5 patients who subsequently developed left ventricular dysfunction had previously exhibited electrocardiographic alterations, further supporting the hypothesis that early repolarization abnormalities may precede measurable impairment in systolic function. It should be acknowledged that the endpoints evaluated in this study differ from the conventional definitions of cancer therapy–related cardiac dysfunction (CTRCD), which are primarily based on LVEF decline and cardiac biomarkers, as adopted in randomized trials such as STOP-CA (15) and PREVENT (16). In contrast, our study focused on early electrocardiographic markers of subclinical cardiotoxicity, which may precede overt systolic dysfunction. This approach reflects a complementary strategy aimed at identifying earlier stages of myocardial injury rather than replacing established clinical endpoints.Beyond overt systolic dysfunction, a key finding of the present study is the strong association between statin therapy and a reduced incidence of early ECG abnormalities. Patients not receiving statins exhibited a markedly higher prevalence of QTa and QTc prolongation as well as T-wave flattening, whereas statin-treated patients largely maintained stable repolarization patterns (31, 37).

The attenuation of ECG abnormalities observed in statin-treated patients aligns with the pathophysiological framework proposed (37, 38) in which QTa prolongation exceeding 10% from baseline or an absolute increase of 30–40 ms constitutes a clinically meaningful warning sign of impending cardiotoxicity. Similarly, Kinoshita et al. (31) identified QTa prolongation and T-wave flattening in precordial leads as among the most sensitive electrocardiographic markers of chronic anthracycline-related myocardial injury. The concordance between our findings and these previous observations (31) supports the use of simple ECG indices as inexpensive and widely accessible tools for early risk stratification in cardio-oncology.

Moreover, all patients who developed electrocardiographic alterations during follow-up were clinically asymptomatic. The identification of these silent electrophysiological changes reinforces the potential value of proactive monitoring strategies and supports the role of preventive interventions aimed at mitigating progression toward overt cardiomyopathy (15, 16, 39).

Importantly, the protective association observed with statin therapy cannot be explained solely by lipid lowering. Although statin-treated patients in our cohort exhibited significantly lower total cholesterol and LDL cholesterol levels, the pleiotropic effects of lipophilic statins likely play a central role. Experimental and clinical studies have demonstrated that statins exert antioxidant, anti-inflammatory, and endothelial-stabilizing effects, which may counteract anthracycline-induced oxidative stress, mitochondrial dysfunction, and electrical remodeling (7, 40). In this context, the preservation of normal ECG repolarization patterns in statin-treated patients suggests a stabilizing effect on myocardial electrophysiology beyond metabolic control.

In particular, lipophilic statins may reduce oxidative stress, preserve mitochondrial integrity, modulate inflammatory signaling pathways, and improve endothelial function, thereby limiting myocardial injury and adverse electrophysiological remodeling. Experimental studies further demonstrated that simvastatin attenuates doxorubicin-induced oxidative cardiotoxicity through inhibition of reactive oxygen species generation and reduction of cardiomyocyte apoptosis (41).

The analysis of atrioventricular conduction further supports the cardiac safety profile of statins in this setting. In our cohort, the PR interval remained stable over time both in statin-treated patients and in the overall population, indicating that neither doxorubicin exposure nor statin therapy was associated with clinically relevant disturbances of atrioventricular conduction. This finding is particularly relevant, as it suggests that the observed differences in ECG abnormalities are specific to ventricular repolarization rather than reflecting a generalized conduction impairment.

The consistency of findings across multiple statistical approaches, including adjusted and propensity-weighted analyses, strengthens the robustness of the observed associations despite the retrospective design and limited sample size.

The concept of frailty deserves particular emphasis. Frail patients, often elderly, multiple comorbidities, and characterized by reduced physiological reserve, are at increased risk of both cardiovascular events and chemotherapy-related complications. In such individuals, therapeutic goals frequently shift from strict lipid target achievement to preservation of functional status and prevention of destabilizing events.

Importantly, our findings extend previous observations by demonstrating a cardioprotective association specifically with simvastatin, corresponding to a moderate-intensity statin, basing on current dosing classifications (42). This suggests that the beneficial effects of statin therapy in anthracycline-treated patients may not be strictly dependent on statin potency, but rather on pleiotropic properties shared across lipophilic statins. This has relevant clinical implications, as it indicates that even lower-intensity statins (at that dosage) such as simvastatin may confer protection, potentially allowing broader applicability in more fragile or elderly populations, where tolerance to high-intensity statin therapy is often limited.

Overall, these findings support a potential cardioprotective role of simvastatin at functional, electrophysiological, and metabolic levels during anthracycline therapy and warrant further prospective evaluation in cardio-oncology settings (42).

## Limitations

Several limitations of this study should be acknowledged.

Firstly, the retrospective design and the relatively small sample size limit causal inference and may contribute to wide confidence intervals in multivariable analyses. Moreover, the treatment protocols may have included medications that could potentially prolong or influence outcomes, the regimens were broadly comparable across patients.

Furthermore, statin therapy was initiated at similar doses among participants; however, owing to the retrospective design of the study, the exact duration of prior statin exposure could not be reliably determined.

In addition, residual confounding cannot be excluded, and cumulative anthracycline dose and duration of statin therapy could not be fully standardized.

An additional limitation of the present study is the absence of serial measurements of established cardiac biomarkers such as troponins and natriuretic peptides (e.g., BNP or NT-proBNP), which are currently recommended for the early detection (HFA-ICOS score) and monitoring of anthracycline-induced cardiotoxicity. The lack of these biomarkers precludes direct comparison between biochemical and electrocardiographic indicators of subclinical myocardial injury.

Moreover, the classification of cardiac dysfunction was based on traditional heart failure criteria (LVEF < 50%) rather than on the more granular definitions of cancer therapy–related cardiac dysfunction proposed by contemporary cardio-oncology guidelines, which incorporate relative declines in LVEF and strain imaging parameters. This may have led to underestimation of subclinical or mild forms of cardiotoxicity.

Furthermore, LVEF assessment was based on standard echocardiographic techniques, which may be subject to inter- and intra-observer variability, particularly in the absence of systematic three-dimensional imaging across all patients.

Additionally, advanced echocardiographic parameters such as LV global longitudinal strain and ventricular volume measurements were not consistently available, limiting the possibility of detecting more subtle forms of subclinical myocardial dysfunction.

Finally, longer follow-up would be required to determine whether the attenuation of early ECG abnormalities translates into a sustained reduction in clinically overt cardiomyopathy.

Taken together, these considerations suggest that the present findings should be interpreted within an exploratory framework focused on early detection of subclinical cardiotoxicity, providing supportive evidence for future prospective studies integrating ECG, imaging, and biomarker-based approaches.

## Conclusions

In this real-world cohort of patients exposed to doxorubicin, treatment with simvastatin was associated with a lower incidence of early repolarization abnormalities and a smaller decline in ventricular function. Electrocardiographic changes, particularly involving ventricular repolarization, appeared to precede overt systolic impairment and were detectable in clinically asymptomatic patients, supporting their role as early markers of anthracycline-related myocardial injury.

These findings highlight the subclinical nature of early cardiotoxicity and support the potential value of simple ECG monitoring as an accessible tool for early risk stratification in cardio-oncology. Notably, the observed protective association with simvastatin, a moderate-intensity statin, suggests that cardioprotective effects may not be strictly dependent on statin potency, thereby supporting its potential use in more vulnerable or frail populations.

Larger prospective studies integrating electrocardiographic, imaging, and biomarker-based approaches are warranted to confirm these findings and to define their place in preventive cardio-oncology care.

## Data Availability

The raw data supporting the conclusions of this article will be made available by the authors, without undue reservation.
