# Supplementary material for: SIMVASTATIN as a potential protective strategy against doxorubicin-induced cardiotoxicity
Source: Front Cardiovasc Med. 2026 Jun 9;13:1846253. doi: 10.3389/fcvm.2026.1846253 (PMC13286747; doi:10.3389/fcvm.2026.1846253)
Supplement: Supplementary file 1 [file Supplementaryfile1.docx]

Supplementary Material

*Propensity Score and Weighted Analyses*

**
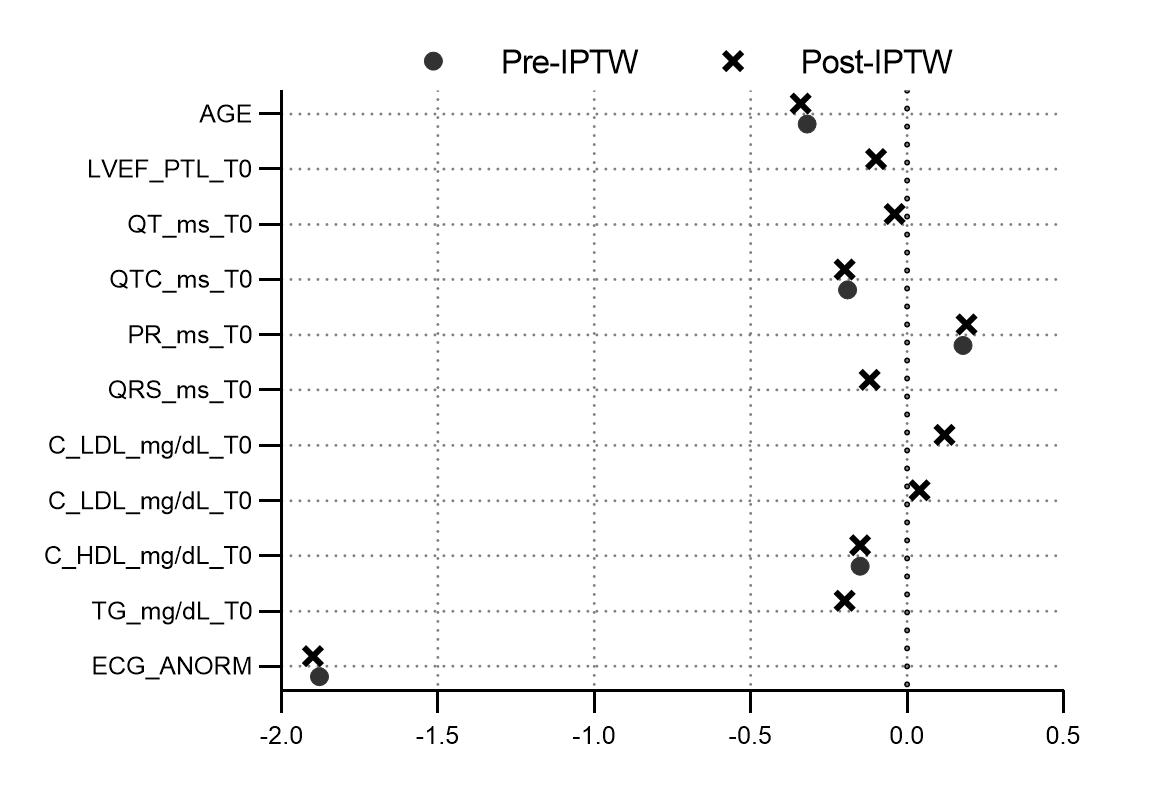
**To address potential confounding due to non-random statin exposure, a propensity score for simvastatin treatment was estimated using baseline demographic, echocardiographic, ECG, and metabolic variables. Inverse probability of treatment weighting (IPTW) substantially improved covariate balance, reducing the mean standardized mean difference from 0.22 to 0.06. Weighted marginal structural models confirmed the cardioprotective association of simvastatin, with effect estimates consistent with unweighted analyses for ΔLVEF%, ΔQT/QTc, and binary ECG endpoints.

**Supplementary Figure 1.** Standardized mean differences (SMD) of baseline covariates before and after inverse probability of treatment weighting (IPTW). Following weighting, all covariates showed substantial reduction in imbalance, with SMDs approaching zero (from 0.22 to 0.06), indicating adequate balance between treatment groups and supporting the validity of causal inference analyses. A threshold of SMD < 0.1 was considered indicative of adequate balance.
